# Bayesian interim analysis for prospective randomized studies: reanalysis of the acute myeloid leukemia HOVON 132 clinical trial

**DOI:** 10.1038/s41408-024-01037-3

**Published:** 2024-03-27

**Authors:** Niek G. van der Maas, Jurjen Versluis, Kazem Nasserinejad, Joost van Rosmalen, Thomas Pabst, Johan Maertens, Dimitri Breems, Markus Manz, Jacqueline Cloos, Gert J. Ossenkoppele, Yngvar Floisand, Patrycja Gradowska, Bob Löwenberg, Gerwin Huls, Douwe Postmus, Francesco Pignatti, Jan J. Cornelissen

**Affiliations:** 1 Department of Hematology, Erasmus Medical Center Cancer Institute, Erasmus University Medical Center, Rotterdam, the Netherlands; 2 Department of Biostatistics, Erasmus MC, Rotterdam, the Netherlands; 3 Department of Epidemiology, Erasmus MC, Rotterdam, the Netherlands; 4 University Hospital, Inselspital, Bern, Switzerland; 5 Swiss Group for Clinical Cancer Research (SAKK), Bern, Switzerland; 6 University Hospital Gasthuisberg, Leuven, Belgium; 7 Ziekenhuis Netwerk Antwerpen, Antwerp, Belgium; 8 University Hospital Zurich, Zurich, Switzerland; 9 Amsterdam UMC, location VUMC, Cancer Center Amsterdam, Amsterdam, the Netherlands; 10 Oslo University Hospital, Oslo, Norway; 11 HOVON Foundation, Rotterdam, the Netherlands; 12 University Medical Center, University Groningen, Groningen, the Netherlands; 13 Oncology and Hematology Office, European Medicines Agency, Amsterdam, the Netherlands

**Keywords:** Randomized controlled trials, Acute myeloid leukaemia

## Abstract

Randomized controlled trials (RCTs) are the gold standard to establish the benefit-risk ratio of novel drugs. However, the evaluation of mature results often takes many years. We hypothesized that the addition of Bayesian inference methods at interim analysis time points might accelerate and enforce the knowledge that such trials may generate. In order to test that hypothesis, we retrospectively applied a Bayesian approach to the HOVON 132 trial, in which 800 newly diagnosed AML patients aged 18 to 65 years were randomly assigned to a “7 + 3” induction with or without lenalidomide. Five years after the first patient was recruited, the trial was negative for its primary endpoint with no difference in event-free survival (EFS) between experimental and control groups (hazard ratio [HR] 0.99, *p* = 0.96) in the final conventional analysis. We retrospectively simulated interim analyses after the inclusion of 150, 300, 450, and 600 patients using a Bayesian methodology to detect early lack of efficacy signals. The HR for EFS comparing the lenalidomide arm with the control treatment arm was 1.21 (95% CI 0.81–1.69), 1.05 (95% CI 0.86–1.30), 1.00 (95% CI 0.84–1.19), and 1.02 (95% CI 0.87–1.19) at interim analysis 1, 2, 3 and 4, respectively. Complete remission rates were lower in the lenalidomide arm, and early deaths more frequent. A Bayesian approach identified that the probability of a clinically relevant benefit for EFS (HR < 0.76, as assumed in the statistical analysis plan) was very low at the first interim analysis (1.2%, 0.6%, 0.4%, and 0.1%, respectively). Similar observations were made for low probabilities of any benefit regarding CR. Therefore, Bayesian analysis significantly adds to conventional methods applied for interim analysis and may thereby accelerate the performance and completion of phase III trials.

## Introduction

Time elapsing between design, completion of patient accrual, and final outcome analysis of prospective randomized clinical trials (RCT) is generally very long, which hampers the rapid approval of drugs for patients with a high or unmet clinical need. To allow for timely access to new therapies, regulatory authorities have permitted drug development strategies other than RCTs, which have increasingly been used in (conditional) approval by the FDA and EMA [1, 2]. Recent FDA evaluation of the Accelerated Approval track highlighted the importance of enhancing quality and efficiency in drug development tracks using prospective comprehensive strategies in order to expedite therapeutic advancements [3].

Prospective phase III RCTs are still pivotal to evaluate the risk-benefit ratio of experimental therapies compared to a well-balanced control group [4–7]. The expected benefit of the experimental treatment is often based on data from earlier phase II studies. RCTs may include interim analyses that focus on toxicity or efficacy endpoints to prevent excessive harm for patients or to stop a study early because of early evidence of benefit or futility. However, the conventional, frequentist approach commonly employed to evaluate these endpoints in clinical trials might be limited by implicit prior assumptions, the need for long-term follow-up to observe the number of events required for final evaluation, and conservative interim stopping rules.

Bayesian statistical methods have been proposed as a tool that might meet these limitations, for example to estimate the maximum tolerated dose of a drug in early phase studies. It may allow for an adaptive conduct of trials by incorporating prior knowledge from historical patients with similar disease and treatment characteristics [8–10]. While Bayesian inference has been well-established in the design of phase I/II studies [11–20], its use in prospective phase III RCTs has been more limited [21–28].

This study aims to retrospectively evaluate how external data as prior knowledge can be used to analyze primary and secondary trial endpoints, and to challenge the original study assumptions at successive interim analyses of an RCT. Therefore, we reanalyzed a prospective phase III trial from the Haematology-Oncology for Adults in the Netherlands (HOVON) and the Swiss Group for Clinical Cancer Research (SAKK) cooperative groups in patients with acute myeloid leukemia (AML) which did not meet its primary endpoint [29], and used a dynamic borrowing approach with Bayesian inference to reinforce the control treatment arm with external data from a previous AML trial.

## Methods

### Study design

**Fig. 1:**
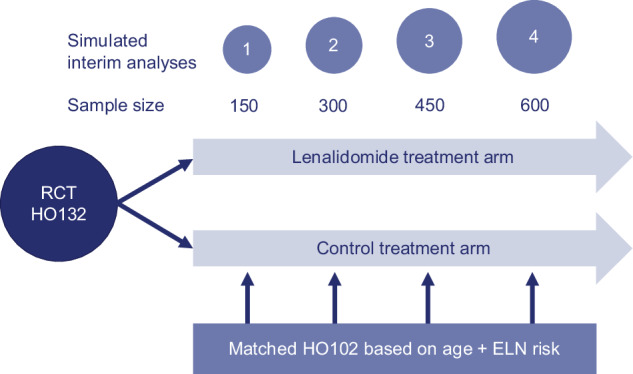
Study design flow diagram. Four interim analyses were simulated in the prospective course of the HO132 after inclusion of 150, 300, 450, and 600 patients. The randomized clinical trial HO132 AML / SAKK 30/13 (HO132) randomized the lenalidomide versus control treatment arm 1:1. A total of 300 control treatment arm patients of the HO132 study and 300 control treatment arm patients of the preceding prospective HOVON 102 AML/SAKK 30/09 (HO102) trial were matched in pairs by propensity scores using age and AML risk (ELN risk) profile [31]. Clinical and outcome data from matched patients included in the HO102 control treatment arm were used to construct a prior for the current control treatment arm of the HO132 in the Bayesian analysis.

In this reanalysis of a randomized phase III clinical trial, the HOVON 132 AML/SAKK 30/13 (HO132) study was used [29]. Four interim analyses were simulated in the prospective conduct of the HO132 trial after the inclusion of 150, 300, 450, and 600 patients for an early benefit-risk assessment (Fig. 1). Outcome data from patients enrolled in the control treatment arm of the preceding prospective HOVON 102 AML/ SAKK 30/09 (HO102) trial [30] were used to reinforce the control treatment arm of the HO132.

### Data sources

Data from the HO132 and HO102 trials were used in this reanalysis (Table 1). The HO132 is a phase III RCT which included patients aged 18 to 65 years with newly diagnosed AML between 2014 and 2017 [29]. Patients were randomized between two cycles of standard remission induction therapy with or without lenalidomide. After remission, induction therapy, patients in complete remission (CR) or CR with incomplete hematologic recovery (CRi) received post‐remission treatment with either a third cycle of chemotherapy, high‐dose chemotherapy followed by autologous stem cell transplantation (SCT) or an allogeneic SCT, as described previously [29]. The primary endpoint was event free survival (EFS), with a total of 800 patients with 441 events being considered to detect an hazard ratio (HR) of 0.76 with 82% power and at the 5% significance level, corresponding with an increased EFS of 10% at 3 years by lenalidomide. Upon final analysis in 2019, EFS was not significantly different between patients receiving intensive induction with or without lenalidomide (HR 0.99, *p* = 0.96) [29]. Additionally, the percentage of patients achieving CR or CRi after two cycles of induction chemotherapy was 82% for the experimental arm and 87% for the control arm (odds ratio [OR] 0.71 *p* = 0.08). Measurable residual disease (MRD) negativity in patients in CR after the second induction cycle were 78 and 77% (OR 0.92, *p* = 0.73), respectively. Early mortality rates at 2 months after the start of treatment were also not different (7 and 5%, respectively). The incidence and severity of adverse events were comparable between the arms during both the induction and maintenance phases, with no evident variations in the frequencies of adverse events.

**Table 1:** Patient outcome and characteristics of studies HO132 & HO102 [29, 30].

	HOVON 132				HOVON 102	
	Lenalidomide treatment arm		Control treatment arm		Historical control treatment arm	
Total	388	100%	392	100%	426	100%
Gender						
Male	233	60.1%	210	53.6%	226	53.1%
Female	155	39.9%	182	46.4%	200	46.9%
Age, years						
Median	54		53		53	
Range	18–65		18–65		18–65	
WBC at diagnosis (x10^9^/l)						
Median	6.7		8.0		6.5	
Range	0–297		0–265		0–341	
ELN risk category^a^						
Favorable	148	38.1%	137	34.9%	147	34.5%
Intermediate	101	26.0%	131	33.4%	113	26.5%
Adverse	139	35.8%	124	31.6%	166	39.0%
CR reached after						
Cycle 1 (early CR)	254	65.5%	276	70.4%	282	66.2%
Cycle 2 (late CR)	65	16.8%	64	16.3%	78	18.3%
Later	30	7.7%	16	4.1%	14	3.3%
Never	39	10.1%	36	9.2%	52	12.2%
MRD						
Negative	201	51.8%	215	54.8%	159	37.3%
Positive	64	16.5%	54	13.8%	67	15.7%
No CR	39	10.1%	36	9.2%	52	12.2%
Missing	84	21.6%	87	22.2%	148	34.7%
Deaths within 60 days	26	6.7%	21	5.4%	34	8.0%
Grade 4–5 adverse events	116	29.9%	112	28.6%	118	27.7%
Overall survival at 4 years (standard error)	55%		54%		44%	
	±3%		±3%		±2%	
Event free survival at 4 years (standard error)	44%		44%		36%	
	±3%		±3%		±2%	
Recruitment period	2014–2017		2014–2017		2010–2013	

*WBC* white blood cell count, *CR* complete remission, *MRD* measurable residual disease.

^a^According to the European LeukemiaNET AML risk classification 2017 by Döhner et al. [31].

The preceding HO102 trial randomized patients between intensive induction treatment with or without clofarabine for patients with newly diagnosed AML, aged between 18 to 65 years. Patients included in the control treatment arm received induction treatment similar to the patients in the HO132 control treatment arm. Patient accrual occurred between 2010 to 2013 and the primary endpoint was EFS.

Propensity score matching between patients from the control treatment arms from both the HO102 and HO132 was used to aim for similar patient characteristics. Propensity scores were obtained through logistic regression on age and European LeukemiaNET 2017 risk [31]. After the propensity score was calculated for each individual, HO102 controls were matched 1:1 to HO132 controls using the nearest neighbor matching algorithm [32]. A total of 300 patients were used from the HO102 control treatment arm, which were matched with HO132 control patients in order to maximize the number of external control patients. Subsequently, outcome data of the 300 patients from the HO102 control treatment arm were used for the construction of the Bayesian prior (see next paragraph, Fig. S1–5) at each interim analysis, meaning that primary analyses were based on the HO132 trial data with a reinforced control treatment arm (for more details see supplementary methods). The median follow-up time was 7, 10, 12 and 16 months at each simulated interim analysis, respectively.

### Bayesian statistical methods

Bayesian inference is a method of statistical inference using Bayes’ theorem to update a probability distribution of a parameter when new information is obtained. Three key concepts need to be considered including (1) the prior distribution (prior), (2) the likelihood, and (3) the posterior probability. The prior is a probability distribution that represents the prior knowledge before seeing any data. The prior can be based on previously observed data or expert opinion. Non-informative priors can be used when no prior data or expert opinion is available. The likelihood is a function that describes the probability density of the newly observed data. The posterior probability is a probability distribution based on the prior distribution combined with the likelihood of newly observed data. The posterior probability represents the updated belief of an event or hypothesis given the available evidence (Fig. S6). Three Markov Monte Carlo chains were run with 50,000 iterations. Chain convergence was evaluated by quantile plots and the Gelman–Rubin diagnostic. See supplementary methods for more details about dynamic borrowing with the commensurate prior.

Posterior probability distributions of the treatment difference (i.e. risk difference) were calculated for efficacy endpoints including (1) EFS, (2) CR rate after two cycles of chemotherapy, (3) proportion of MRD negative patients in CR after cycle 2, and safety endpoints including (4) rate of early mortality within 2 months after start of treatment, and (5) rate of grade 4–5 adverse events. The probability distributions were summarized to provide point estimates of the probability distribution median and 95% Bayesian credible intervals (95% CI) on the treatment difference and HR between both arms. CR, MRD, adverse events above grade III and early death outcomes were assessed with Bayesian beta-binomial models and the probability of any benefit (treatment difference > 0%) of the lenalidomide treatment versus the control treatment arm was estimated. EFS was evaluated using a Bayesian Weibull survival model and the probability distributions of the HR for EFS were used to estimate the probability of HR < 0.76, which was the assumed effect size for EFS in the HO132 study. Less optimistic treatment effects were also studied, including the probability of EFS HR < 0.87, which corresponded to a 5% increase in EFS at 3 years by lenalidomide. Lastly, the probability of EFS HR < 1 was estimated, which corresponds to any benefit in EFS for the lenalidomide treatment arm.

All Bayesian analyses were performed in R version 4.2.2 with the additional software of JAGS, using the package “rjags” [33, 34]. The R script of the analyses can be found online (https://github.com/niekvandermaas/Bayesian_reanalysis_HO132_paper).

### Conventional futility methods

In this study, a conventional group sequential design was retrospectively implemented to monitor early efficacy and futility through four interim analyses at the same time points as for the Bayesian approach with EFS as the primary endpoint. This sequential design was based on the original HO132 statistical analysis plan and would require 883 patients with 488 events to detect a HR of 0.76 with 82% power and a one-sided Type I error of 2.5%. The higher number of patients and events in a group sequential design reflects the penalty of four interim efficacy analyses. Efficacy and futility bounds were derived using a Lan-DeMets O’Brien-Fleming approximation spending function, and the analysis was conducted using EAST statistical software [35].

## Results

### Benefit-risk assessment at interim analyses

#### Treatment efficacy: EFS

**Fig. 2:**
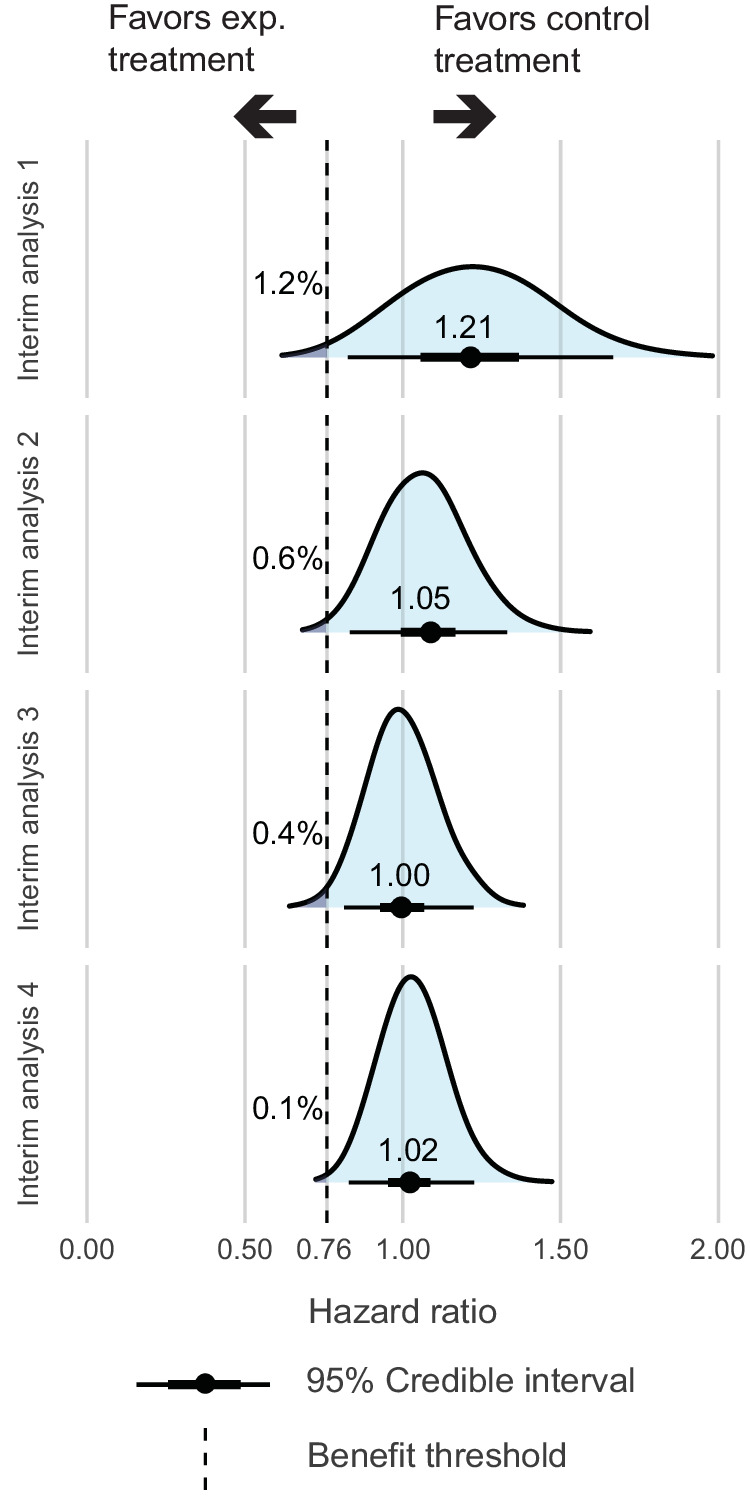
Estimation of the probability for the assumed treatment effect (HR < 0.76) by Bayesian analysis of event free survival comparing lenalidomide treatment versus reinforced control treatment. The blue bell shape in the figure depicts the posterior distribution of the HR between arms for event free survival with the median HR. The benefit threshold is set at the assumed treatment effect of HR = 0.76. The arrows on left of the benefit threshold indicates the probability for HR < 0.76, showing the evidence for the assumed benefit of the experimental treatment arm and right of the benefit threshold the probability for HR > 0.76.

Lenalidomide treatment was compared with the reinforced control treatment at the four defined interim time-points. The HR for EFS was 1.21 (95% CI 0.81 to 1.69), 1.05 (95% CI 0.86 to 1.30), 1.00 (95% CI 0.84 to 1.19), and 1.02 (95% CI 0.87 to 1.19), at interim analyses 1, 2, 3, and 4, respectively (Fig. 2, Table S1). At interim analyses 1 and 2, the probability of being below the anticipated HR of 0.76 was 1.2% and 0.6%, which probability was 0.4% at interim analyses 3 and 0.1% at interim analysis 4 (Fig. 2, Table S1). The probability for a moderate treatment benefit in EFS (HR < 0.87) at interim analysis 1, 2, 3, and 4 was 5.0%, 6.5%, 9.0%, and 4.4%, respectively (Fig. S7). The probability of any benefit (HR < 1.0) for the lenalidomide treatment arm compared with the control treatment arm was moderate at all interim analyses, with a probability for any benefit of 16.4%, 32.9%, 49.0%, and 41.0%, respectively (Fig. S8).

**Fig. 3:**
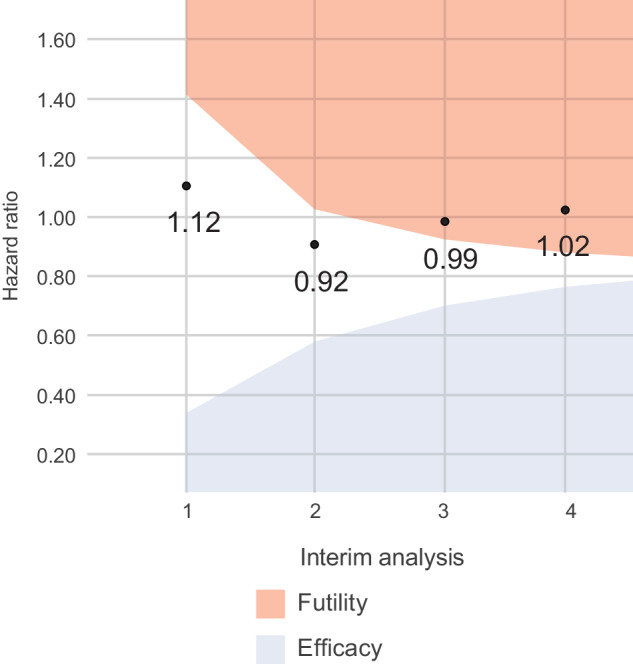
Conventional group sequential design with non-binding two-sided hazard ratio bounds for stopping for efficacy or futility at each interim analysis. The conventional group sequential design with non-binding two-sided hazard ratio bounds for stopping for efficacy or futility at each interim analysis. The red area in the figure is the futility boundary. If an observed HR lies within the red area, the study may be stopped for futility. The blue area in the figure is the efficacy boundary. Similarly, if an observed HR lies within the blue area, the study may be stopped for efficacy. The estimated event-free survival hazard ratio for lenalidomide treatment effect was 1.12, 0.92, 0.99, and 1.02 at interim analysis 1, 2, 3, and 4, respectively.

While the lack of treatment efficacy for EFS was already identified early after the first interim analysis using Bayesian inference with 150 patients enrolled, a conventional group sequential design showed that the HR of treatment benefit by lenalidomide crossed the futility boundary at the third interim analysis with 450 patients randomized (Fig. 3). The observed HR for EFS comparing the lenalidomide arm with the control treatment arm was 1.12, 0.92, 0.99, and 1.02 at interim analysis 1, 2, 3, and 4 respectively (Fig. 3).

#### Treatment efficacy: CR and MRD negativity

**Fig. 4:**
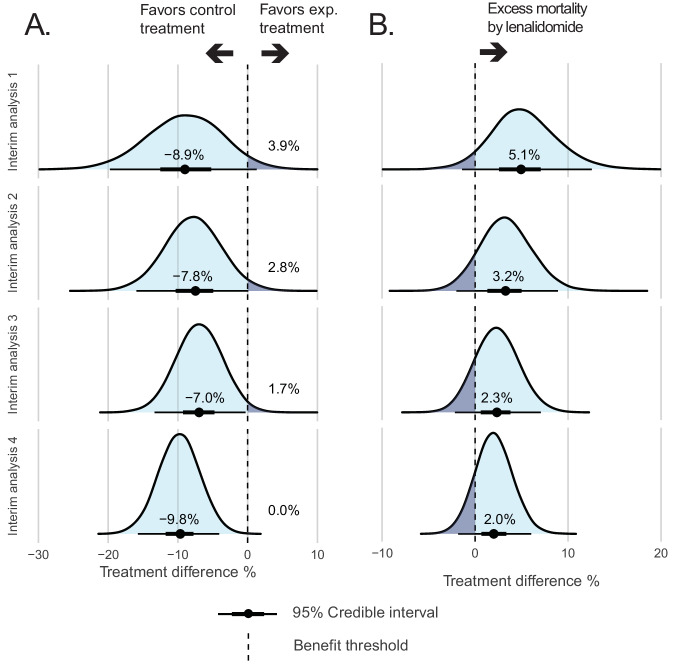
Comparison of lenalidomide treatment versus reinforced control treatment by Bayesian analysis of CR and early death rate. The blue bell shape in the figure depicts the posterior distribution of the treatment difference between arms for CR after two cycles of chemotherapy (**A**) or early death rate (**B**) with the median treatment difference. The benefit threshold is set at no difference or zero.

In the lenalidomide treatment arm, the median percentage of patients obtaining CR was lower compared with the control treatment arm at interim analysis 1 (82% vs 91% comparing the lenalidomide arm vs control treatment arm; treatment difference: −8.9%; 95% CI −19.9 to 1.0, Fig. 4A, Table S1) and the probability of a higher CR rate in the lenalidomide treatment arm compared with the control treatment arm was 3.9% (Fig. 4A, Table S1). At interim analyses 2 to 4 the median CR proportions resulted in treatment differences of −7.8% (79% vs 87%; 95% CI −16.0 to 0.02), −7.0% (80% vs 87%; 95% CI −13.5 to −0.5), and −9.8% (78% vs 88%; 95% CI −15.6 to −4.1) with a probability of a higher CR rate of 2.8%, 1.7%, and 0.0%, respectively (Fig. 4A, Table S1). A low probability of a higher CR rate in the lenalidomide treatment arm suggests no benefit of this treatment compared with the control treatment arm.

Data on MRD after two induction cycles in patients who obtained a CR was available in 78.1% of patients, which was equally balanced between treatment arms (Table 1). Patients in CR and assigned to the lenalidomide treatment arm were less often MRD negative compared with the control treatment arm at every interim analysis. At interim analysis 1, 73% of patients in the lenalidomide treatment arm were in MRD negative CR, whereas 83% of patients were MRD negative in the control treatment arm (treatment difference: −10.3%; 95% CI −28.5 to 6.8, Fig. S9, Table S1). The probability for a higher MRD negative CR rate for the lenalidomide treatment arm compared with the control treatment arm was 12.2% at the first interim analysis. At interim analyses 2 to 4, the probability of higher MRD negative rates were 10.1%, 6.2%, and 12.0%, respectively (Fig. S9, Table S1). Similar to CR, a low likelihood of a higher MRD rate in the lenalidomide treatment arm indicates that this treatment does not have any advantage compared with the control treatment arm.

#### Treatment toxicity: early death and adverse events

Death within the first 60 days of treatment was more frequently observed in the experimental treatment arm compared with the control treatment arm at interim analysis 1 (7% vs 2%; treatment difference: 5.1%; 95% CI −1.3 to 12.8, Fig. 4B, Table S1). The treatment differences at interim analyses 2 to 4 were 3.2% (8% vs 5%; 95% CI −2.0 to 9.1), 2.3% (8% vs 6%; 95% CI −2.2 to 7.2), and 2.0% (8% vs 6%; 95% CI −1.9 to 6.1), respectively. Grade 4–5 adverse events were not different between both arms in any of the interim analyses (Fig. S10, Table S1).

### Interim analyses without external data

The impact of the external data (HO102 trial) on the primary outcome analysis of EFS for lenalidomide vs control treatment was determined by performing a Bayesian analysis without external data (Table S2). The HR was 1.09 (95% CI 0.72 to 1.65), 0.95 (95% CI 0.71 to 1.28), 1.00 (95% CI 0.78 to 1.27), and 1.05 (95% CI 0.85 to 1.29) at interim analyses 1 to 4, respectively (Table S2), which corresponds to a similarly low probability of the assumed benefit (HR < 0.76) for EFS of 4.7%, 7.0%, 1.6%, and 0.2%, respectively (Table S2). This suggests that the external data had a relatively limited impact on the Bayesian analysis at interim time points. The probability of a moderate treatment effect (HR < 0.87) for EFS in favor of the lenalidomide treatment arm was 14.8%, 27.5%, 14.5%, and 4.4%, respectively and for any benefit by lenalidomide for EFS was 34.7%, 62.7%, 52.6%, and 33.6%.

## Discussion

A prospective RCT is the preferred type of trial to evaluate the benefits and risks of new therapies [4–7]. Historical reports have highlighted that 71% of RCTs in hemato-oncology resulted in non-significant outcomes or negative findings. This observation might be linked to unrealistically high expectations regarding the treatment effect size [36]. Currently, innovative approaches are being developed that may accelerate and enhance the knowledge arising from prospective studies, including phase II and phase III studies. Bayesian statistical methods have been applied in phase I/II studies but may also be applied in phase III RCTs. Here, we retrospectively simulated four interim analyses within a recent phase III RCT, randomizing patients for lenalidomide in AML [29]. We evaluated outcome parameters during patient accrual using a Bayesian approach and compared it to conventional frequentist statistical methods. At all four Bayesian interim analyses, the likelihood of the expected benefit (HR < 0.76) was very low. In contrast, the frequentist group sequential design declared futility in the third interim analysis. Additionally, our Bayesian analysis of efficacy endpoints, including CR and MRD negative CR, at four interim time points showed a low probability of benefit (<15%) by lenalidomide. Risk assessment showed excess mortality in patients randomized to the lenalidomide arm. Our data indicate that interim analyses in phase III clinical trial using Bayesian inference addressing both the benefits and risks of an experimental drug proved to be highly informative.

Randomized phase III clinical trials are generally designed, taking into account results from earlier phase I/II studies. By virtue of randomization, the experimental treatment can be evaluated to a concurrent control population with little or minimal bias. Bayesian statistics additionally allow for the use of external data in the context of a RCT, next to the randomized control population. It may be done so upon trial completion, but also during patient accrual at specific interim analysis time points. It might allow for a reinforced control treatment arm with an informative prior [8]. External and current data need to be carefully matched for risk factors and eligibility criteria to avoid selection bias as much as possible. Here, a previous trial conducted by HOVON-SAKK was used, that included patients with similar inclusion criteria and control treatment. It enabled a rapid and complete matching procedure by a dynamic borrowing approach, which improved the precision of the posterior probabilities for EFS. In addition, it might be recommended to consider the results with and without added prior knowledge in order to investigate the impact of the external data. Here, a low probability of obtaining an HR < 0.76 for EFS was also observed without the external control treatment data, suggesting that these data did not essentially change the conclusion of the Bayesian analysis at interim time points. The external data added relatively limited value because the lenalidomide treatment arm performed worse than the control treatment arm for multiple efficacy endpoints, including EFS, CR and MRD negative CR, already from an early time point. Although the conclusion of the interim analyses were not different using external data, reinforcing a control arm increases statistical power, which consequently may potentially lead to a reduction in sample size [37]. Furthermore, Bayesian analysis at interim time points may provide broader-based recommendations to an independent safety and monitoring board (DSMB) of an ongoing study, which should preferably remain blinded to the trial team. If an alarming signal at multiple interim analyses time points arises, that may impact the advice to the principal investigator and trial team. However, to implement a Bayesian sequential design in the future, it has been recommended by regulatory authorities to evaluate the operating characteristics such as power and type I error rate, for establishing a futility threshold [38].

A Bayesian approach to interim analysis might have several limitations. First, Bayesian approaches or adaptive designs cannot control for selection bias and residual confounding, highlighting the importance of a control arm in a randomized setting. Second, incorporating overly positive (or negative) prior information may introduce bias impacting the posterior distribution [39]. Third, we assumed that clinical data from the HO102 trial were comparable to the HO132 trial. While there were no large differences in baseline characteristics between the two studies, EFS was significantly different without matching. After matching, EFS at interim analysis was similar illustrating that without addressing changes in the underlying population by e.g. matching methods, external data may introduce bias.

RCTs are often criticized for their limited generalizability due to the selection of patients, such as the exclusion of patients with older age and comorbidities [40, 41]. Real-world data (RWD) contain patient health and healthcare data of patients, predominantly outside the context of clinical trials [42]. Thereby, these data have the potential to provide insights into the benefits and risks of therapeutic interventions in a more generalizable patient population. Similar as done in this study with external data, RWD may be applied in the context of prospective phase III studies, in order to reinforce the control population. Nevertheless, an ongoing challenge is to approximate the quality of RWD and trial data as much as possible [43].

In conclusion, simulations of four interim analyses in the HO132 study showed that the assumed benefit of lenalidomide was unlikely to be achieved, which was already observed after the first interim analysis using Bayesian inference with external data as an informative prior, whereas a conventional evaluation would have a futility conclusion only after the third interim analysis. These results augment conventional futility analyses, highlighting the potential of Bayesian statistical methods to provide earlier and highly informative insights into trial outcomes at interim time points. This methodology might be considered to expedite clinical trial adaptation and enhance efficiency in drug development. External data, such as historical trial data or RWD, may be used to increase the precision of the control treatment arm of clinical trials, but caution must be taken to ensure data comparability and minimize bias.

### Supplementary information

Supplementary Materials

## Data Availability

The data that support the findings of this study are available from HOVON but restrictions apply to the availability of these data, which were used under license for the current study, and so are not publicly available. Data are, however, available from the authors upon reasonable request and with permission of HOVON.
