# Supplementary material for: Bayesian interim analysis for prospective randomized studies: reanalysis of the acute myeloid leukemia HOVON 132 clinical trial
Source: Blood Cancer J. 2024 Mar 27;14(1):56. doi: 10.1038/s41408-024-01037-3 (PMC10973506; doi:10.1038/s41408-024-01037-3)
Supplement: Supplementary file 1 — Supplementary Materials [file 41408_2024_1037_MOESM1_ESM.pdf]

SUPPLEMENTARY MATERIALS

Contents

Supplementary materials....................2

Supplementary methods....................2

Figure S1 Bayesian prior of control treatment EFS log hazard rate. 3

Figure S2 Bayesian prior of control treatment complete remission rate. 4

Figure S3 Bayesian prior of control treatment MRD negative CR rate. 5

Figure S4 Bayesian prior of control treatment early death rate. 6

Figure S5 Bayesian prior of control treatment adverse events grade 4-5 rate. 7

Figure S6 Bayesian inference example. 8

Figure S7 Event free survival HR<0.87. 9

Figure S8 Event free survival HR<1.00. 10

Figure S9 Minimal residual disease negativity in CR patients. 11

Figure S10 Adverse events grade 4-5. 12

Table S1 Results of the interim-analyses. 13

Table S2 Results of the interim-analyses without external data. 14

References 15

SUPPLEMENTAL METHODS

Bayesian inference is a method of statistical inference using Bayes theorem to update a probability distribution of a parameter when new information is obtained. Three key concepts need to be considered including (1) the prior distribution (prior), (2) the likelihood, and (3) the posterior probability. The prior is a probability distribution that represents the prior knowledge before seeing any data. The prior can be based on previously observed data or expert opinion. Non-informative priors can be used when no prior data or expert opinion is available. The likelihood is the probability density of the newly observed data. The posterior probability is a probability distribution based on the prior distribution combined with the likelihood of newly observed data. The posterior probability represents the updated belief of an event or hypothesis given the available evidence (Figure S6).

The commensurate prior is a Bayesian approach for dynamic borrowing (downweighing of information) proposed by Hobbs et al., which has been extended by incorporating a spike and slab prior.[1] This method centers the priors for the current data's model parameters on the corresponding parameters for the historical data, and assumes a distribution for the difference in model parameters between the current and historical data. Spike-and-slab prior distributions are used to model the variances of these parameters for the current data, conditional on the parameters of the historical data.[2]

The follow-up times were censored at 60 days after the start of the second chemotherapy cycle of the last enrolled HO132 patient in the respective interim analysis. To ensure a comparable follow-up time with the HO132 (maximum follow-up time, 10, 16, 22, 28 months at interim analysis 1, 2, 3 and 4 cut-off, respectively), an analysis window from date of registration to the maximum follow-up time was applied to the HO102 control treatment arm at each simulated interim analysis, respectively. The median follow-up time was 7, 10, 12 and 16 months at each simulated interim analysis, respectively.

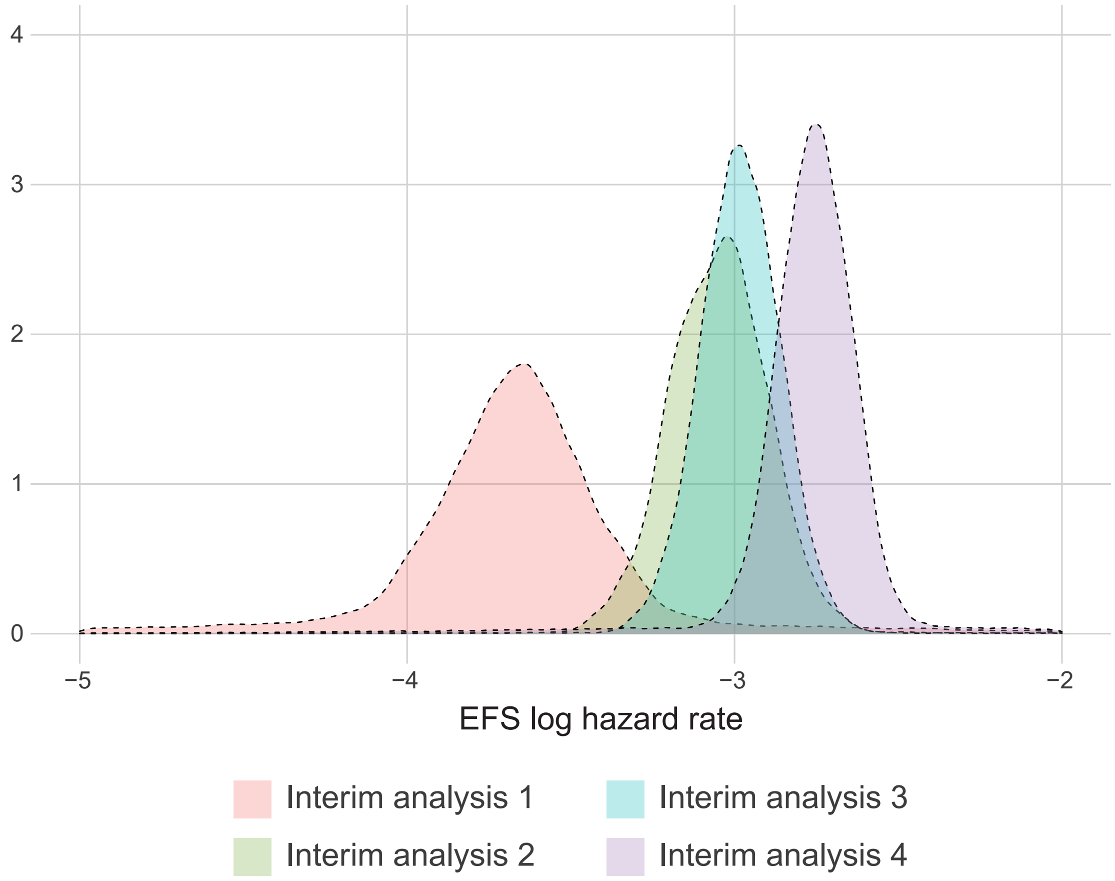

Figure S1 Priors of HO132 control treatment arm EFS log hazard rate.

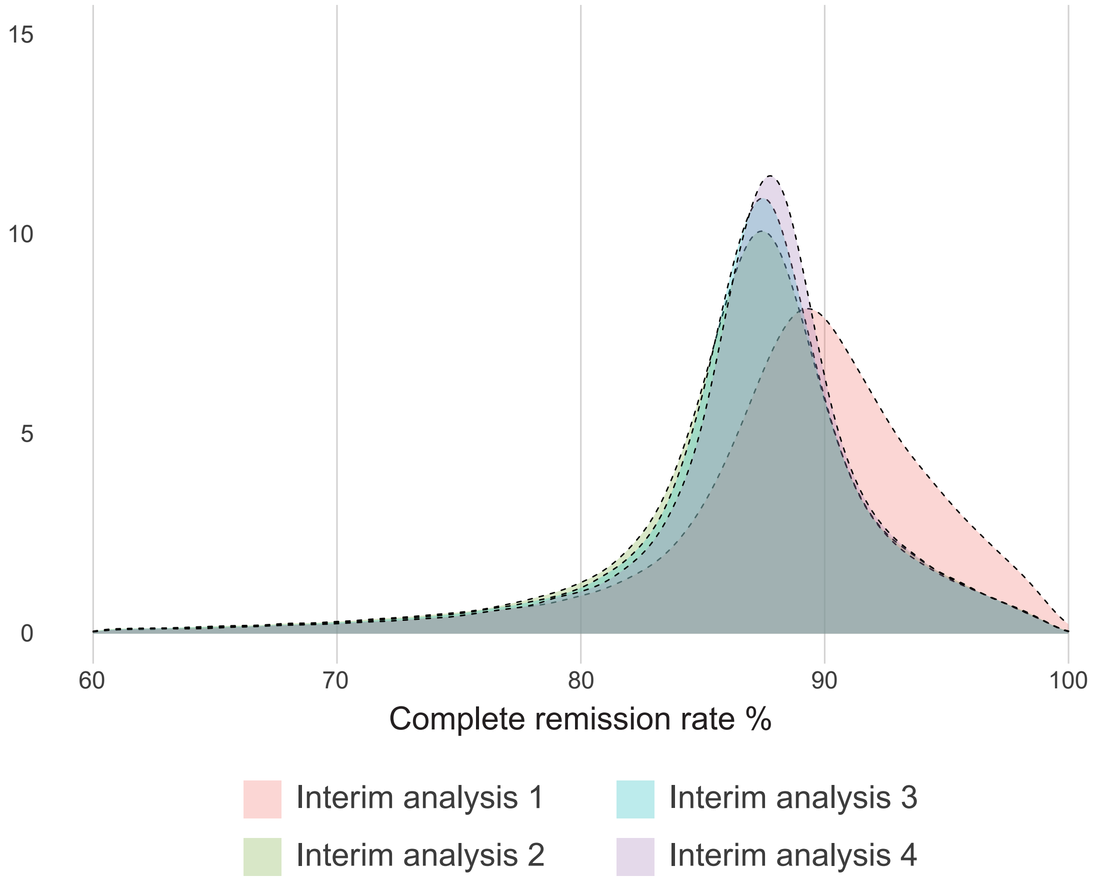

Figure S2 Priors of HO132 control treatment arm complete remission rate.

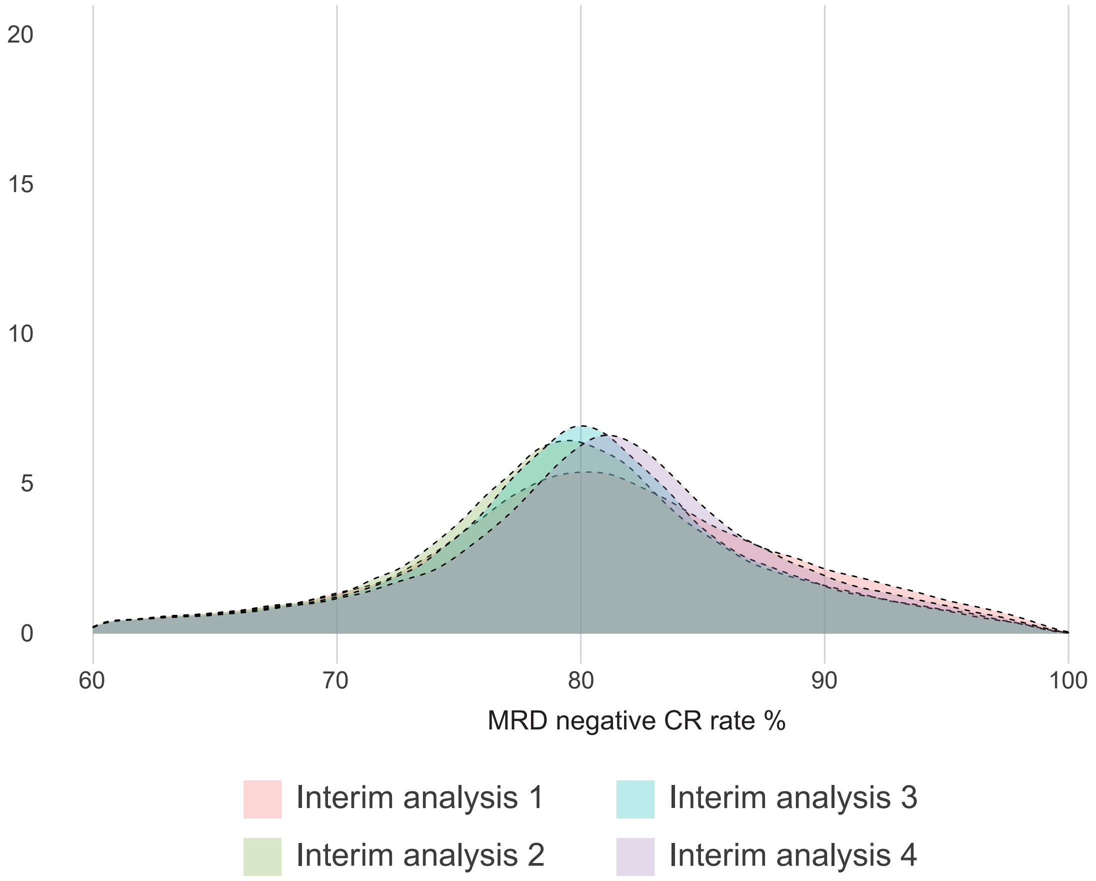

Figure S3 Priors of HO132 control treatment arm MRD negative CR rate.

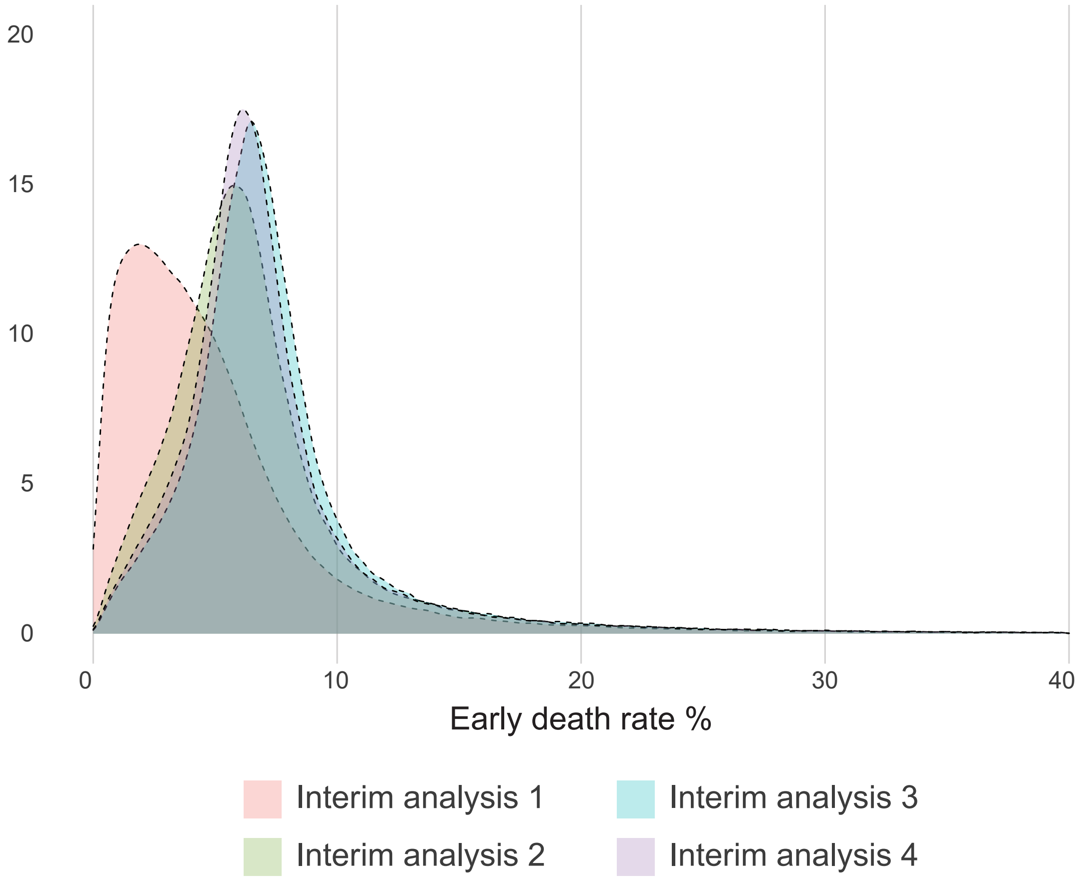

Figure S4 Priors of HO132 control treatment arm early death rate.

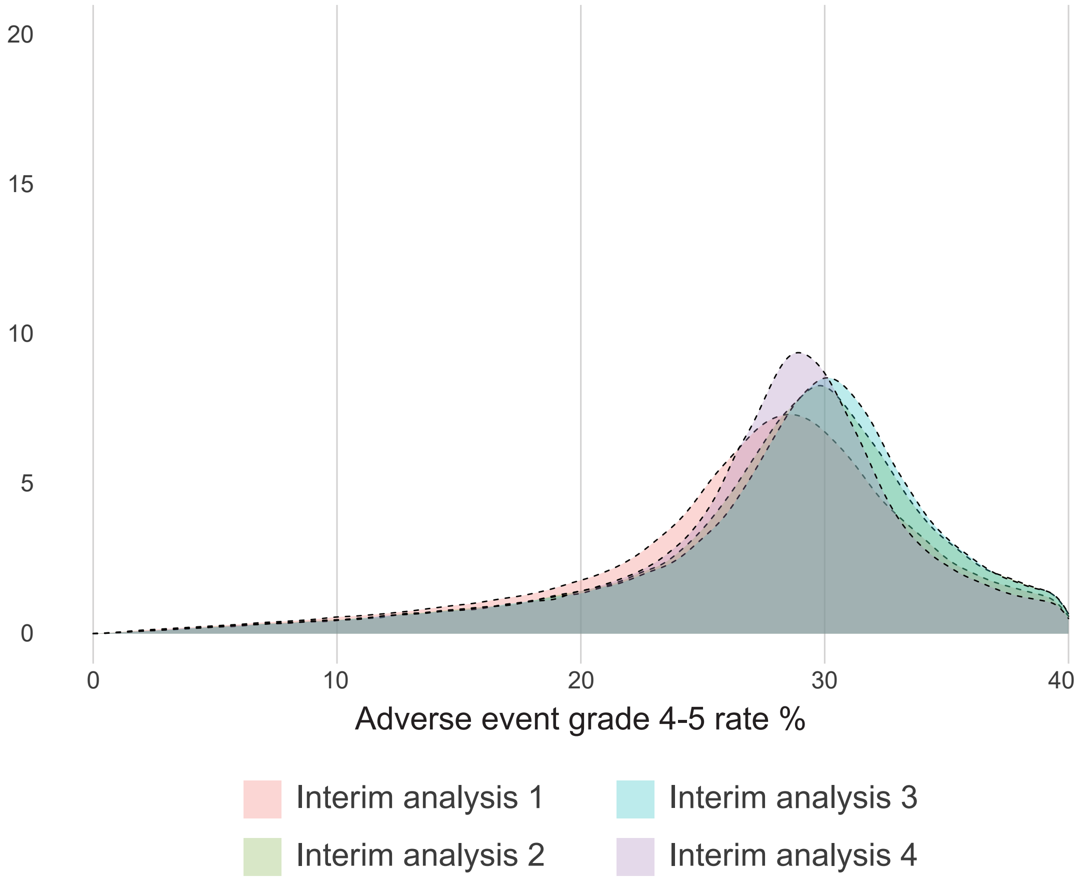

Figure S5 Priors of HO132 control treatment arm adverse event grade 4-5 rate.

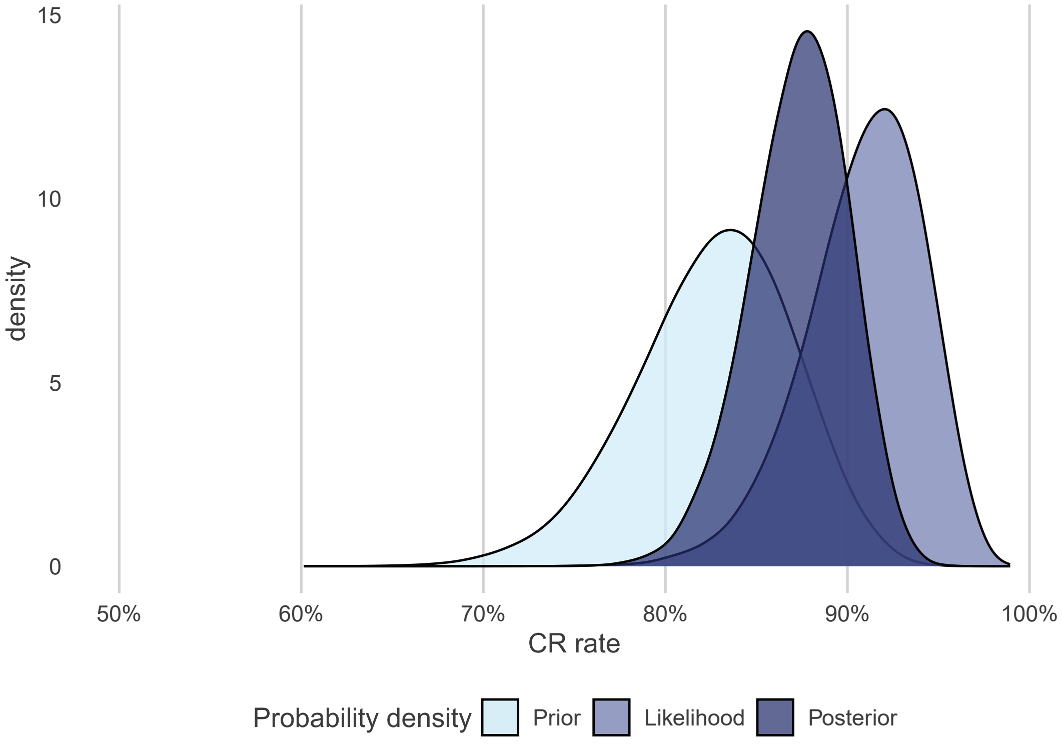

Figure S6 Bayesian inference example.

Bayesian inference is a method of statistical inference using Bayes theorem to update a probability distribution of a parameter when new information is obtained. Three key concepts need to be considered including (1) the prior distribution (prior), (2) the likelihood, and (3) the posterior probability. The prior is a probability distribution that represents the prior knowledge before seeing any data. The prior can be based on previously observed data or expert opinion. Non-informative priors can be used when no prior data or expert opinion is available. The likelihood is the probability density of the newly observed data. The posterior probability is a probability distribution based on the prior distribution combined with the likelihood of newly observed data. The posterior probability represents the updated belief of an event or hypothesis given the available evidence.

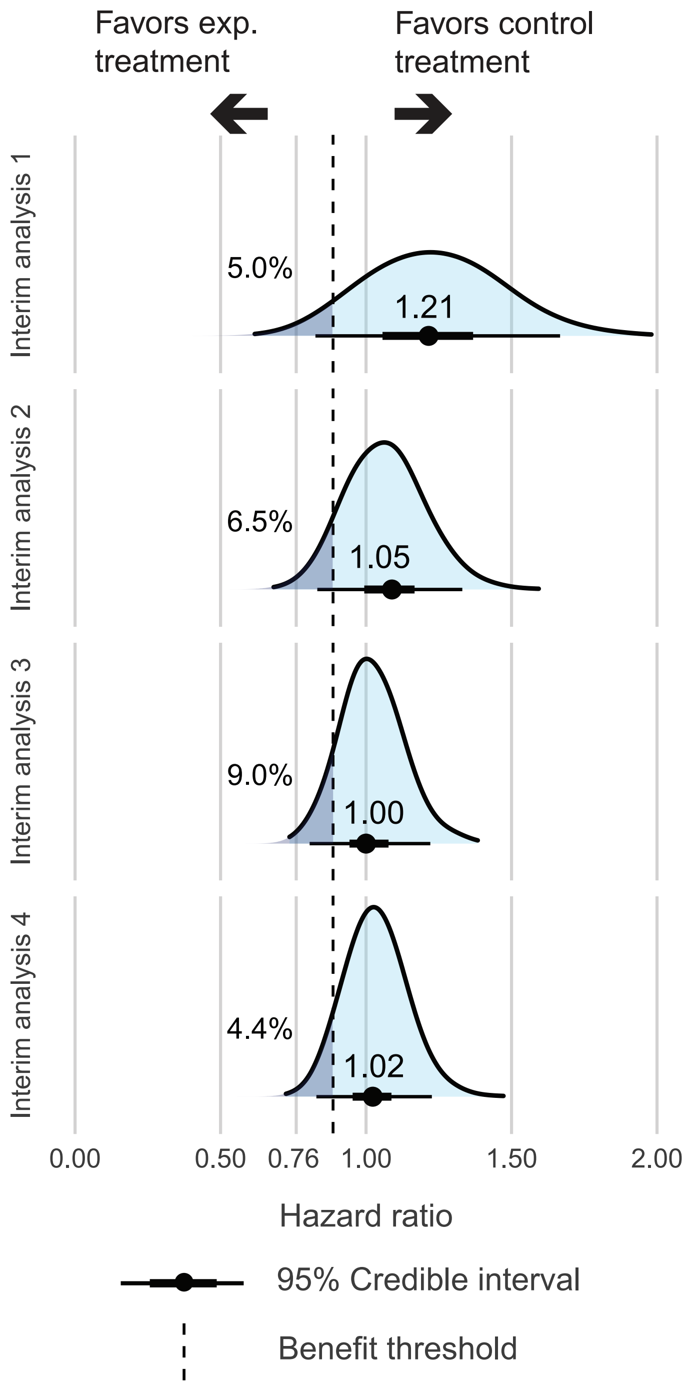

Figure S7 Estimation of the probability for specified treatment (HR<0.87) effects by Bayesian analysis of event free survival comparing lenalidomide treatment versus control treatment with informative prior.

The blue bell shape in the figure depicts the posterior distribution of the HR between arms for event free survival with the median HR. The benefit threshold is set at the assumed treatment effect of HR=0.87. The arrows indicate left of the benefit threshold the probability for HR<0.87, showing the evidence for the targeted benefit of the experimental treatment arm and right of the benefit threshold the probability for HR>0.87.

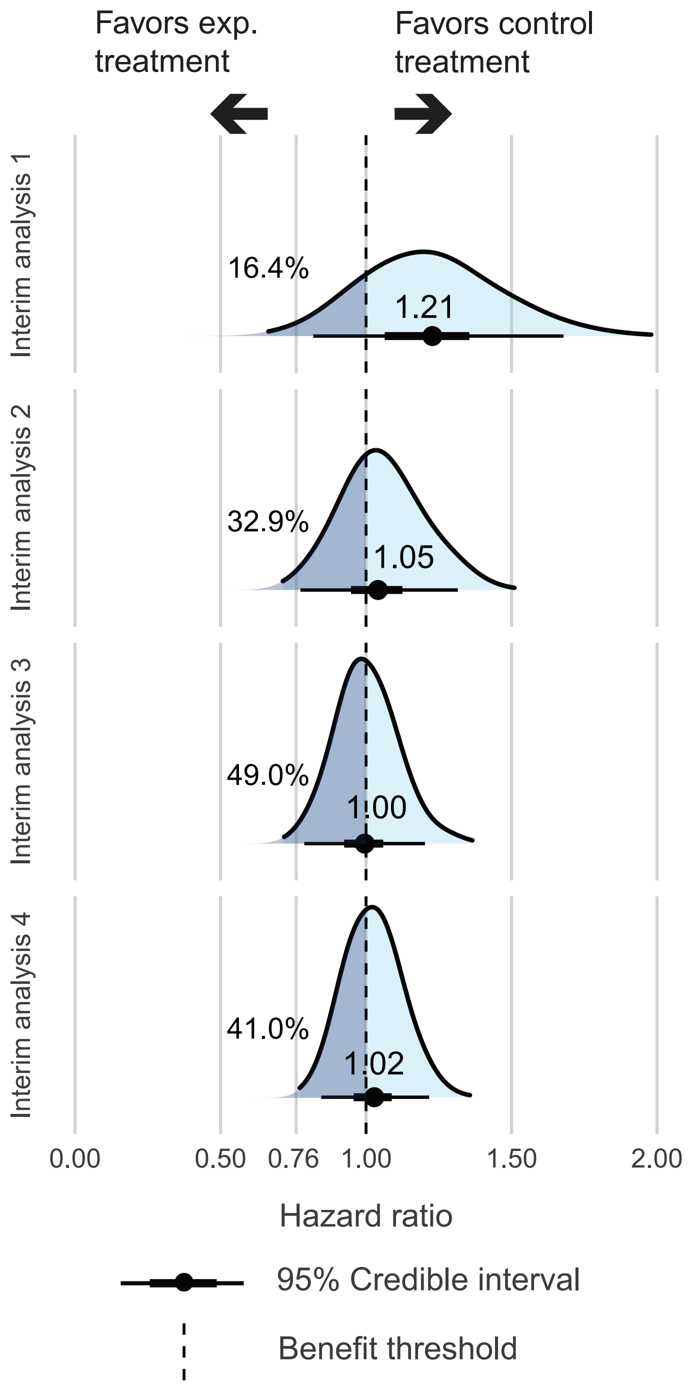

Figure S8 Estimation of the probability for specified treatment (HR<1.00) effects by Bayesian analysis of event free survival comparing lenalidomide treatment versus control treatment with informative prior.

The blue bell shape in the figure depicts the posterior distribution of the HR between arms for event free survival with the median HR. The benefit threshold is set at the assumed treatment effect of HR=1.00. The arrows indicate left of the benefit threshold the probability for HR<1.00, showing the evidence for the targeted benefit of the experimental treatment arm and right of the benefit threshold the probability for HR>1.00.

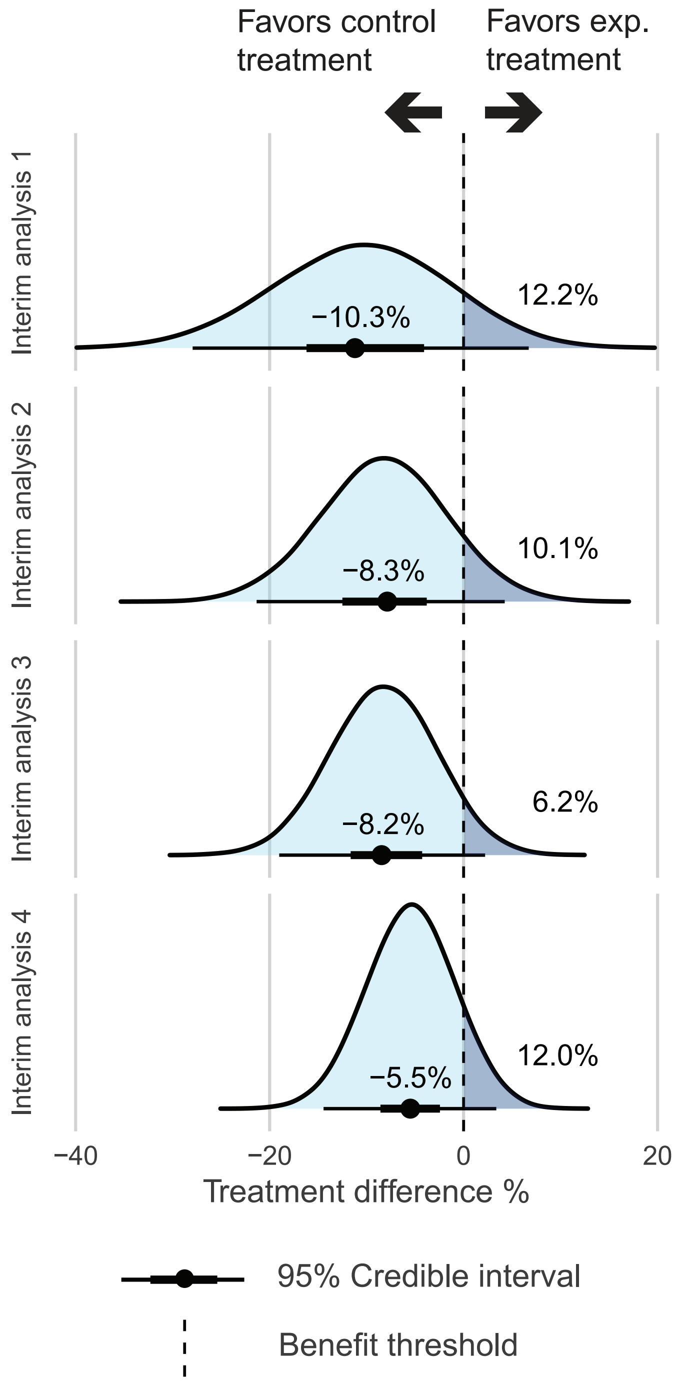

Figure S9 Comparison of lenalidomide treatment versus control treatment by Bayesian analysis of MRD negative patients in CR with informative prior.

The blue bell shape in the figure depicts the posterior distribution of the treatment difference between arms for MRD negative patients in CR with the median treatment difference. The benefit threshold is set at no difference or zero. The arrow to the left of the benefit threshold indicates less MRD negativity in the lenalidomide treatment arm compared to the control treatment arm, thus showing the evidence favoring the control treatment arm. The arrow to the right of the benefit threshold indicates more MRD negativity in the lenalidomide treatment arm compared to the control treatment arm, thus showing the evidence favoring the lenalidomide treatment arm. A negative median indicates less MRD negativity in the lenalidomide treatment arm compared to the control treatment arm.

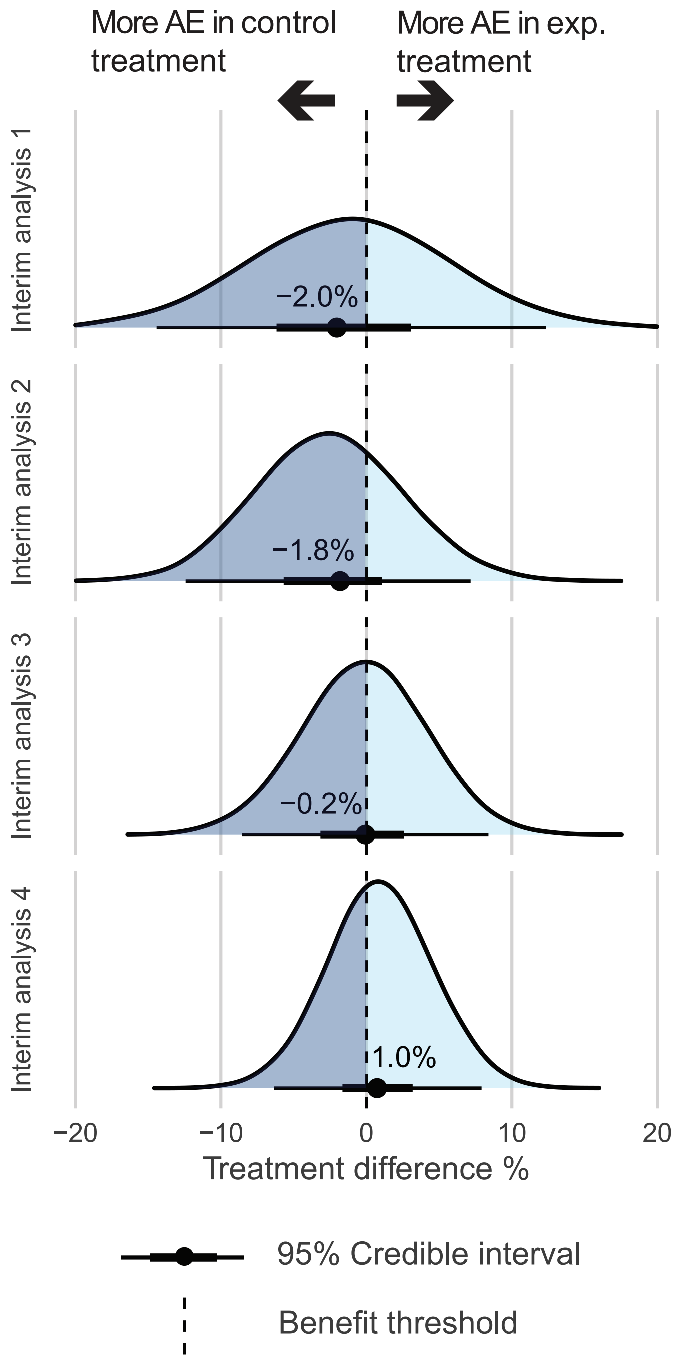

Figure S10 Comparison of lenalidomide treatment versus control treatment by Bayesian analysis of adverse events grade 4-5 with informative prior.

The blue bell shape in the figure depicts the posterior distribution of the treatment difference between arms for AE grade 4-5 with the median treatment difference. The benefit threshold is set at no difference or zero. The arrow to the left of the benefit threshold indicates less AE in the lenalidomide treatment arm compared to the control treatment arm, showing the evidence favoring the control treatment arm. The arrow to the right of the benefit threshold indicates more AE in the lenalidomide treatment arm compared to the control treatment arm, showing the evidence favoring the lenalidomide treatment arm.

Table S1 Results of the interim-analyses.

	Interim-analysis 1 (n=150)			Interim-analysis 2 (n=300)		
	**Median**	**95% CI**		**Median**	**95% CI**	
**Hazard ratio**			**Pr<0.76**			**Pr<0.76**
EFS	1.21	0.81 to 1.69	1.2%	1.05	0.86 to 1.30	0.6%
**Treatment difference**			**Pr>0**			**Pr>0**
CR	-8.9%	-19.9 to 1.0	3.9%	-7.8%	-16.0 to 0.02	2.8%
MRD^{neg}	-10.3%	-28.5 to 6.8	12.2%	-8.3%	-21.2 to 4.3	10.1%
Early deaths	5.1%	-1.3 to 12.8		3.2%	-2.0 to 9.1	
AE grade 4-5	-2.0%	-15.0 to 11.4		-1.8%	-11.8 to 8.1	

	Interim-analysis 3 (n=450)			Interim-analysis 4 (n=600)		
	**Median**	**95% CI**		**Median**	**95% CI**	
**Hazard ratio**			**Pr<0.76**			**Pr<0.76**
EFS	1.00	0.84 to 1.19	0.4%	1.02	0.87 to 1.19	0.1%
**Treatment difference**			**Pr>0**			**Pr>0**
CR	-7.0%	-13.5 to -0.5	1.7%	-9.8%	-15.6 to -4.1	0.0%
MRD^{neg}	-8.2%	-19.0 to 2.2	6.2%	-5.5%	-14.3 to 3.5	12.0%
Early deaths	2.3%	-2.2 to 7.2		2.0%	-1.9 to 6.1	
AE grade 4-5	-0.2%	-8.6 to 8.0		1.0%	-6.2 to 8.0	

Abbreviations: 95% CI, 95% credible interval; Pr, probability; CR, complete remission; MRD^{neg}, minimal residual disease negativity in CR; AE, adverse events; EFS, event free survival.

Table S2 Results of the interim-analyses without external data.

	Interim-analysis 1 (n=150)			Interim-analysis 2 (n=300)		
	**Median**	**95% CI**		**Median**	**95% CI**	
**Hazard ratio**			**Pr<0.76**			**Pr<0.76**
EFS	1.09	0.72 to 1.65	4.7%	0.95	0.71 to 1.28	7.0%
**Treatment difference**			**Pr>0**			**Pr>0**
CR	-9.5%	-20.4 to 0.9	3.7%	-7.5%	-15.9 to 1.0	4.2%
MRD^{neg}	-11.9%	-29.8 to 6.5	10.0%	-9.1%	-22.1 to 4.3	9.2%
Early deaths	5.6%	-0.1 to 13.1		3.6%	-1.9 to 9.4	
AE grade 4-5	-2.4%	-16.5-11.8		-2.5%	-12.8 to 7.9	

	Interim-analysis 3 (n=450)			Interim-analysis 4 (n=600)		
	**Median**	**95% CI**		**Median**	**95% CI**	
**Hazard ratio**			**Pr<0.76**			**Pr<0.76**
EFS	1.00	0.78 to 1.27	1.6%	1.05	0.85 to 1.29	0.2%
**Treatment difference**			**Pr>0**			**Pr>0**
CR	-6.8%	-13.6 to 0.0	2.5%	-9.7%	-15.7 to -3.8	0.0%
MRD^{neg}	-8.8%	-19.6 to 1.9	5.4%	-5.9%	-14.9 to 2.9	9.3%
Early deaths	2.4%	-2.5 to 7.3		2.1%	-1.9 to 6.2	
AE grade 4-5	-0.8%	-9.4 to 7.8		0.7%	-6.6 to 8.1	

Abbreviations: 95%CI, 95% credible interval; Pr, probability; CR, complete remission; MRD^{neg}, minimal residual disease negativity; AE, adverse events; EFS, event free survival. Priors used were Jeffreys prior for binary outcomes and a non-informative normal prior with mean zero and precision (1/variance) 1.0E-6 for the survival outcome.

REFERENCES

1. Hobbs BP, Sargent DJ, Carlin BP. Commensurate Priors for Incorporating Historical Information in Clinical Trials Using General and Generalized Linear Models. Bayesian Anal. 2012;7(3):639–74.

2. Mitchell TJ, Beauchamp JJ. Bayesian Variable Selection in Linear Regression. J Am Stat Assoc 1988;83(404):1023–1032.
